# Application of fluorescence methods for characterization of ligand binding to G protein coupled receptors

**DOI:** 10.1186/2193-1801-4-S1-L19

**Published:** 2015-06-12

**Authors:** Ago Rinken, Anni Allikalt, Sergei Kopanchuk, Reet Link, Santa Veikšina

**Affiliations:** Institute of Chemistry, University of Tartu, Estonia; Institute of Chemistry, University of Tartu, Estonia, Competence Centre on Health Technologies, Tartu, Estonia

**Keywords:** GPCR, Fluorescence anisotropy, budded baculoviruses

G protein coupled receptors (GPCR) comprise a large family of transmembrane proteins involved in the regulation of signal transduction through the cell membrane in response to various extracellular stimuli. GPCR have become important targets of many drugs for treatments of very different diseases. During the last decade several fluorescence-based methods have been implemented for the characterization of signal transduction via GPCRs, starting from ligand binding and including several steps leading up to a response on the level of gene regulation. We have proposed the fluorescence anisotropy (FA) and fluorescence intensity (FI) assay to investigate fluorescent ligand binding properties to different GPCRs (Veiksina et al., 2010). The implementations of budded baculoviruses that display G protein-coupled receptors on their surfaces have significantly increased sensitivity and applicability of these assays (Veiksina et al., 2014). The developed novel assay systems opened new possibilities for real-time monitoring of ligand binding to their receptors for understanding their particular kinetic properties. These assays are also compatible for homogenous HTS suitable fo ligand screening. There has been implemented assay systems for receptors of peptides like melanocortin (MC4R) and neuropeptide Y (NPY1R) as well as for receptors of monoamines like dopamine (D1DAR) and serotonin (5-HT1AR).

